# Diffuse Infantile Hepatic Hemangiomatosis: A Rare Cause of Consumptive Hypothyroidism

**DOI:** 10.7759/cureus.108137

**Published:** 2026-05-02

**Authors:** Karima Larbi Ouassou, Azzeddine Laaraje, Radi Abdelilah, Hassani Amale, Rachid Abilkassem

**Affiliations:** 1 Pediatric Medicine, Mohammed V Military Training Hospital, Rabat, MAR; 2 Pediatric Critical Care, Mohammed V Military Training Hospital, Rabat, MAR

**Keywords:** consumptive hypothyroidism, hepatic mri, infant, infantile hepatic hemangiomatosis, propranolol

## Abstract

Infantile hepatic hemangiomatosis (IHH) is a rare benign vascular tumor of infancy, defined by multiple angiomatous lesions diffusely replacing the hepatic parenchyma. Its clinical course can be severe due to systemic complications, particularly consumptive hypothyroidism and high-output heart failure. We report the case of a four-month-old female infant admitted for abdominal distension evolving over two weeks. Physical examination revealed massive hepatomegaly (hepatic span of 14 cm) with abdominal-thoracic collateral venous circulation and no cutaneous hemangiomas. Laboratory workup showed normochromic normocytic non-regenerative anemia (hemoglobin = 6.7 g/dL), hepatic cytolysis (aspartate aminotransferase = 3× normal), cholestasis (alkaline phosphatase = 188 IU/L, gamma-glutamyl transferase = 335 IU/L), and a prothrombin time reduced to 54%. Alpha-fetoprotein was 470 ng/L. Abdominal MRI revealed multiple rounded hepatic lesions, T1 hypointense and T2 hyperintense with diffusion restriction and progressive centripetal contrast enhancement, consistent with diffuse IHH. Cardiac (echocardiography) and cerebral (transfontanellar ultrasound) assessments were normal. Systematic thyroid function tests revealed severe consumptive hypothyroidism, with thyroid-stimulating hormone level of 500 µIU/mL and free T4 of 6 pmol/L, without antithyroid antibodies. Management included progressive propranolol (Hemangiol®) up to 3 mg/kg/day, levothyroxine at 20 µg/kg/day, and red blood cell transfusion. At the six-month follow-up, thyroid function had normalized, and hepatic lesions showed significant regression. This case highlights the necessity of systematic thyroid function screening in all infants with IHH and illustrates the efficacy of propranolol in managing this rare but potentially life-threatening condition.

## Introduction

Infantile hemangioma (IH) is the most common benign tumor of infancy, occurring in approximately 4-10% of children under one year of age, with a marked female predominance (ratio of 2.5 to 4 girls per boy) [1,2]. According to the International Society for the Study of Vascular Anomalies (ISSVA) classification, IH is a benign vascular tumor, formally distinct from vascular malformations, characterized by postnatal anarchic proliferation of immature endothelial cells, whose specific histological marker is the expression of the glucose transporter GLUT-1 [3].

From a pathophysiological standpoint, antenatal or perinatal hypoxic mechanisms are believed to induce overexpression of vascular endothelial growth factor (VEGF) and basic fibroblast growth factor (bFGF), thereby triggering the endothelial proliferation characteristic of these tumors [4]. Well-established predisposing factors include female sex, fair skin, prematurity (affecting up to 25% of infants weighing less than 1,500 g), multiple gestations, and perinatal hypoxia [1,5].

In the vast majority of cases, IHs are cutaneous and follow a spontaneously favorable course with gradual regression over several years [6,7]. Visceral involvement is nonetheless possible, with the liver representing the most common extracutaneous site after the upper airways [1,2]. Infantile hepatic hemangiomatosis (IHH) differs from isolated hepatic hemangioma by its multifocal nature, its capacity to replace nearly the entire hepatic parenchyma, and the potential severity of its systemic complications [8,9].

These complications include high-output heart failure secondary to intratumoral arteriovenous shunting, consumptive hypothyroidism, severe anemia, and, more rarely, hepatic rupture [8,10]. Consumptive hypothyroidism, a poorly recognized yet characteristic complication of IHH, results from excessive and irreversible degradation of thyroid hormones by type 3 iodothyronine deiodinase (ID3) overproduced by hemangiomatous tissue. This enzyme catalyzes the inactivation of thyroxine (T4) into biologically inactive reverse T3 (rT3) and triiodothyronine (T3) into inert diiodothyronine (T2), inducing a state of profound hypothyroidism proportional to tumor burden [8,9].

The therapeutic revolution initiated by the landmark 2008 discovery by Léauté-Labrèze of the efficacy of propranolol in IH [11] profoundly transformed the management of these tumors. This non-cardioselective beta-blocker, whose anti-angiogenic action involves reduction of VEGF secretion, immediate vasoconstriction, and induction of endothelial cell apoptosis, is now the first-line treatment for complicated IH, with a pediatric oral solution (Hemangiol®) receiving marketing authorization in 2014 [6,7,11].

We report the case of a four-month-old infant presenting with diffuse IHH revealed by abdominal distension, complicated by severe consumptive hypothyroidism, and successfully treated with combined propranolol and levothyroxine therapy, with favorable six-month follow-up.

## Case presentation

Initial presentation

The patient was a four-month-old female infant from southern Morocco, born of a non-consanguineous union. There was no notable personal, neonatal, or family history. The infant was admitted to our pediatric department for the investigation of progressive abdominal distension evolving over two weeks before admission. On admission, general clinical examination found a conscious, reactive, and tonic infant with satisfactory growth parameters and hemodynamically, neurologically, and respiratorily stable.

Physical examination

Abdominal examination revealed marked abdominal distension with an umbilical circumference of 50 cm, visible abdominal-thoracic collateral venous circulation, and significant hepatomegaly with a hepatic span of 14 cm. No splenomegaly or palpable mass was noted. Skin and mucosal examination was notable for the complete absence of purpuric lesions and cutaneous hemangiomas. The remainder of the somatic examination was unremarkable.

Laboratory workup

Initial laboratory investigations are summarized in Table 1. Findings revealed normochromic normocytic non-regenerative anemia (hemoglobin = 6.7 g/dL), mean corpuscular volume of 79.9 fL, mean corpuscular hemoglobin of 33.2 pg, and reticulocyte count of 80,000/mm³. The full blood count was otherwise normal. Liver function tests showed moderate cytolysis with aspartate aminotransferase three times the upper limit of normal, together with biochemical cholestasis (alkaline phosphatase = 188 IU/L, gamma-glutamyl transferase = 335 IU/L). Coagulation studies revealed a prothrombin time reduced to 54% with a normal activated partial thromboplastin time. Renal function, serum electrolytes, and calcium-phosphorus balance were within normal limits. Urinary catecholamines were negative, ruling out pheochromocytoma or neuroblastoma. Alpha-fetoprotein (AFP) was 470 ng/L with beta-human chorionic gonadotropin (β-hCG) below 2.3 µg/L, excluding a malignant germ cell tumor, while remaining consistent with benign hepatopathy at this age.

**Table 1:** Summary of laboratory investigations.

Parameters	Patient values	Reference range
Hematology		
Hemoglobin (g/dL)	6.7	9.5–14.0 (infant)
Mean corpuscular volume (fL)	79.9	70–86
Mean corpuscular hemoglobin (pg)	33.2	23–31
Reticulocyte count (/mm³)	80,000	20,000–100,000
White blood cell count (/mm³)	Normal	5,000–15,000
Platelet count (/mm³)	Normal	150,000–450,000
Liver function tests		
Aspartate aminotransferase (U/L)	3× upper limit of normal	<40
Alkaline phosphatase (IU/L)	188	<350 (infant)
Gamma-glutamyl transferase (IU/L)	335	<45
Coagulation		
Prothrombin time (%)	54	70–100
Activated partial thromboplastin time	Normal	Normal
Tumor markers		
Alpha-fetoprotein (ng/L)	470	Physiologically elevated in infancy
Beta-human chorionic gonadotropin (µg/L)	<2.3	<5
Urinary catecholamines	Negative	Negative
Thyroid function tests		
Thyroid-stimulating hormone (µIU/mL)	500	0.5–5.0
Free T4 (pmol/L)	6	10–26
Anti-thyroid peroxidase antibodies	Negative	Negative
Anti-thyroglobulin antibodies	Negative	Negative
Renal function and metabolic panel		
Renal function	Normal	Normal
Serum electrolytes	Normal	Normal
Calcium-phosphorus balance	Normal	Normal

Imaging

**Figure 1:**
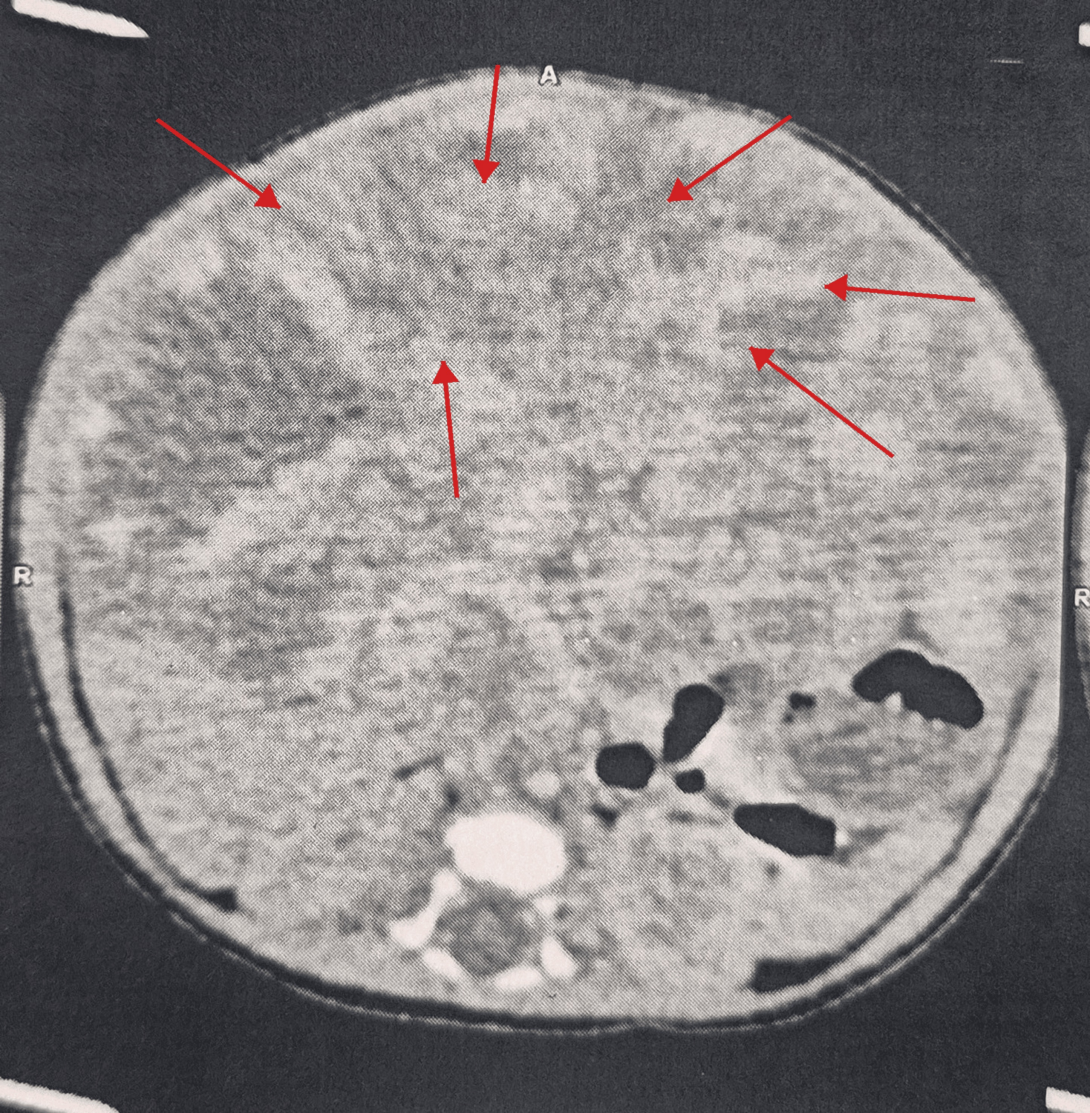
Transverse abdominal CT scan showing diffuse hepatomegaly. The red arrows indicate multiple hypervascular nodular lesions scattered throughout the hepatic parenchyma, consistent with diffuse infantile hepatic hemangiomatosis.

Initial abdominal ultrasound demonstrated a liver suggestive of chronic hepatopathy, with hepatomegaly at 14.7 cm and heterogeneous pseudonodular parenchyma. Abdominal CT (Figure 1) confirmed extensive hepatomegaly reaching the right iliac fossa, with a heterogeneous hepatic appearance and multiple contiguous lesions.

**Figure 2:**
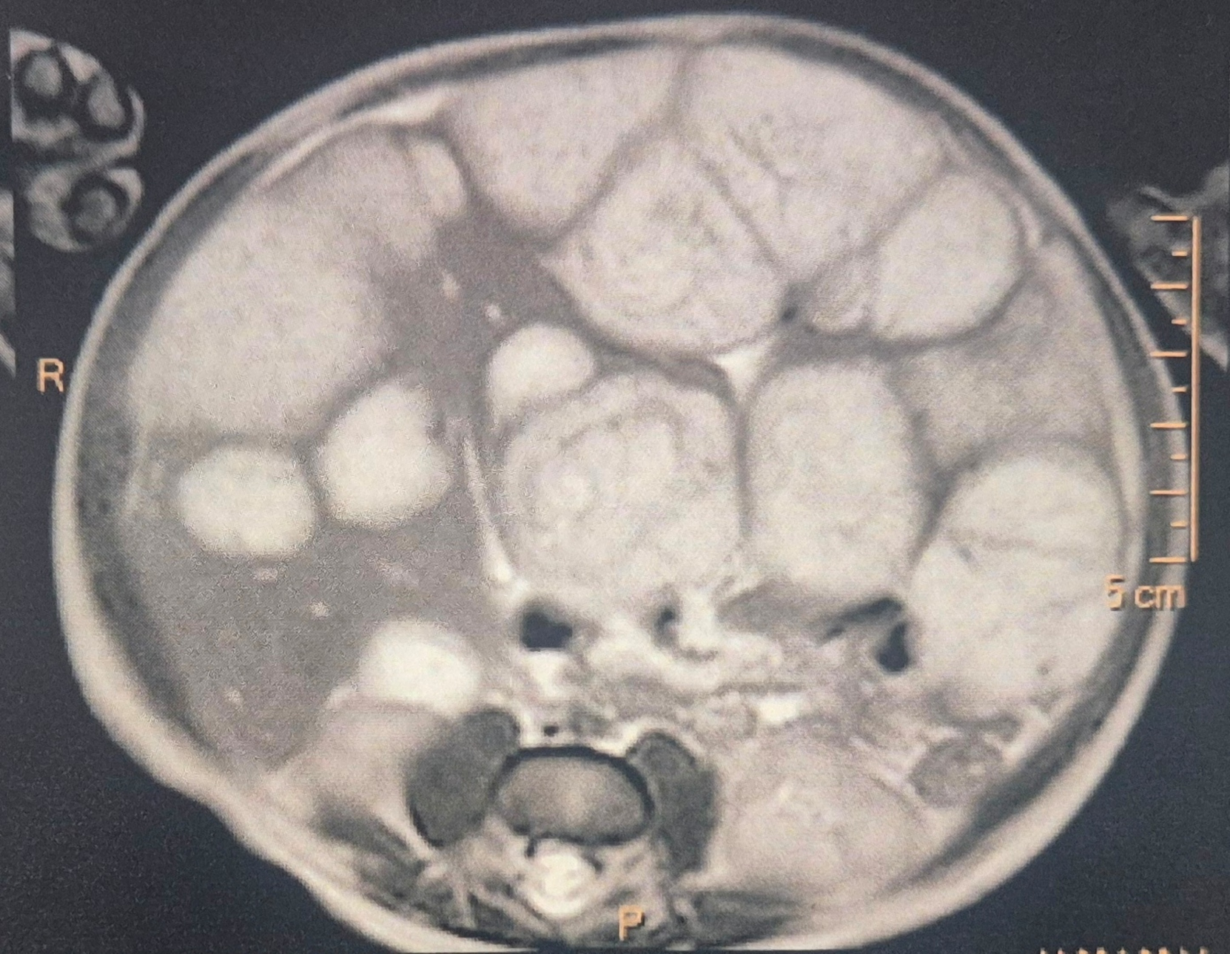
Abdominopelvic MRI showing evidence of infantile hepatic hemangiomatosis.

Abdominopelvic MRI (Figure 2), the gold standard for characterization of focal hepatic lesions in children, demonstrated hepatomegaly with multiple rounded lesions, T1-hypointense and T2-hyperintense, containing necrotic areas with diffusion restriction, and showing progressive centripetal enhancement following gadolinium injection. The largest lesions measured 50 × 40 mm in segment V and 40 × 35 mm in segment VIII. This radiological pattern was highly consistent with diffuse IHH.

Transthoracic echocardiography was normal, revealing no heart failure or intracardiac shunt. Transfontanellar ultrasound, performed to screen for extrahepatic hemangiomatosis, was likewise normal.

Thyroid function assessment

In accordance with current management guidelines for IHH, systematic thyroid function tests were performed. These revealed severe hypothyroidism: thyroid-stimulating hormone (TSH) of 500 µIU/mL (normal < 5 µIU/mL) and free T4 of 6 pmol/L (normal = 10-26 pmol/L). Anti-thyroid peroxidase (anti-TPO) and anti-thyroglobulin (anti-Tg) antibodies were negative, ruling out autoimmune thyroiditis. Cervical ultrasound was not performed in our patient due to limited resources at the time of initial management; however, the negativity of antithyroid antibodies and the clinical context were sufficient to confirm the peripheral consumptive nature of the hypothyroidism. Taken together, these findings were consistent with consumptive hypothyroidism secondary to excessive production of ID3 by the hepatic hemangiomatous tissue.

Management and outcome

The therapeutic strategy for our patient comprised three complementary components. For the treatment of hemangiomatosis, propranolol (Hemangiol®) was administered at progressive doses with regular monitoring of heart rate and blood glucose, according to the following protocol: 1 mg/kg/day in two divided doses during the first week, 2 mg/kg/day during the second week, and 3 mg/kg/day during the third week. This dosing schedule, consistent with marketing authorization recommendations, was designed to minimize the risk of adverse effects (bradycardia, hypoglycemia, bronchospasm).

For the treatment of consumptive hypothyroidism, levothyroxine (LEVOTHYROX®) was introduced at progressive doses up to 20 µg/kg/day, substantially higher than doses typically used for congenital hypothyroidism (approximately 10-15 µg/kg/day), given the continuous degradation of thyroid hormones by tumoral ID3.

Red blood cell transfusion was performed for symptomatic severe anemia, with a post-transfusion control hemoglobin of 9.7 g/dL.

Clinical and biological outcome at six months was favorable. Thyroid function progressively normalized under levothyroxine therapy. Control hepatic MRI at six months demonstrated a significant reduction in the size of hepatic hemangiomas, confirming the efficacy of propranolol on tumor activity. No notable adverse effects were observed during follow-up.

## Discussion

Epidemiology and pathophysiology

IHH is a rare entity, yet it accounts for approximately 90% of hepatic vascular anomalies in children [10]. According to published data, infantile hepatic hemangiomas are diagnosed as solitary lesions in 40% of cases and as multifocal lesions in 60% of cases [9]. The diffuse form, as observed in our patient, is the least frequent but the most severe. The female predominance classically observed for cutaneous IH (ratio of 2.5 to 4 girls per boy) [1,2] is also observed for hepatic forms.

From a pathophysiological perspective, IHH results from anarchic proliferation of GLUT-1-positive immature endothelial cells, driven by pro-angiogenic factors (VEGF, bFGF) whose overexpression is triggered by antenatal or perinatal hypoxic conditions [4]. The absence of cutaneous hemangiomas in our patient, though frequent in isolated hepatic forms, is noteworthy, as it may delay diagnosis in the absence of cutaneous warning signs.

Clinical presentation and diagnostic characteristics

The clinical presentation of IHH is variable, ranging from an asymptomatic abdominal mass to life-threatening forms. The most frequent presenting signs are abdominal distension, hepatomegaly, and signs of cardiac failure [8-10]. In our observation, massive hepatomegaly with abdominal distension was the revealing sign, without cutaneous involvement or associated heart failure.

The absence of cutaneous hemangiomas in our case is notable. According to the literature, cutaneous hemangiomas are associated with hepatic forms in only 20-40% of cases [1,7]. Conversely, miliary cutaneous hemangiomatosis, defined as more than five cutaneous hemangiomas, warrants a systematic abdominal ultrasound to screen for hepatic involvement [6,7].

The moderate AFP elevation at 470 ng/L warrants discussion. In neonates and young infants, normal AFP values are physiologically very high in the first months of life (potentially exceeding 100,000 ng/L at birth, then progressively declining). A moderate AFP elevation in this context is compatible with benign hepatopathy and should not raise suspicion of malignancy, particularly given negative β-hCG and characteristic clinico-radiological findings [9].

Role of imaging in diagnosis

The diagnosis of IHH relies primarily on imaging. Abdominal ultrasound is the first-line examination, enabling the detection of lesions and orientation toward their vascular nature. It demonstrates ill-defined hepatic masses with a pseudonodular appearance that can be difficult to differentiate from adjacent parenchyma in highly diffuse forms [12,13].

CT confirms the extent of hepatic involvement and allows assessment of lesion vascularity, but MRI is the gold standard for characterization of hepatic hemangiomas in children [12-14]. MRI offers the best diagnostic performance through its combination of complementary sequences: marked T1 hypointensity and T2 hyperintensity (comparable to cerebrospinal fluid signal) reflecting the liquid content of lesions, absence of diffusion coefficient (ADC) restriction (values exceeding those of healthy hepatic parenchyma), and, above all, the characteristic progressive centripetal enhancement pattern following gadolinium injection [12,13]. This peripheral nodular enhancement with progressive centripetal fill-in reflects the histological architecture of cavernous hemangioma, composed of large vascular spaces with slow filling. In highly diffuse forms with confluent lesions, the typical peripheral enhancement pattern may be absent, and only delayed acquisitions are informative [13,14].

IHH must be distinguished from other pediatric hepatic lesions that may enter into the differential diagnosis, including metastatic neuroblastoma (excluded by urinary catecholamine assay), hepatoblastoma (typically associated with marked AFP elevation, often exceeding 10,000 ng/L), and epithelioid hemangioendothelioma (distinguished by its intermediate biological behavior and histological criteria) [8,15]. Liver biopsy, although providing definitive histological confirmation through demonstration of GLUT-1 endothelial expression, is not routinely recommended when imaging is typical and clinical context concordant [8].

Consumptive hypothyroidism: a key complication of IHH

Consumptive hypothyroidism represents a well-recognized but still underdiagnosed complication of IHH [8,9]. It results from overexpression by hemangiomatous tissue of ID3, an enzyme normally present in fetal tissues. This enzyme converts T4 into biologically inactive rT3 and T3 into inert T2, producing accelerated degradation of circulating thyroid hormones. The result is profound hypothyroidism, proportional to tumor burden, refractory to standard replacement doses, and correctable only by supra-physiological doses of levothyroxine [8].

In our observation, a TSH of 500 µIU/mL attests to the depth of hypothyroidism, with a markedly suppressed free T4 of 6 pmol/L. Negativity of antithyroid antibodies (anti-TPO and anti-Tg) rules out autoimmune etiology and confirms the peripheral consumptive nature of the hypothyroidism. Although a cervical ultrasound was not performed in our patient, this limitation does not affect the diagnosis, as the combination of severely elevated TSH, suppressed free T4, negative antithyroid antibodies, and the presence of massive hepatic hemangiomatosis is sufficient to establish consumptive hypothyroidism with certainty.

Untreated hypothyroidism in infancy is a diagnostic and therapeutic emergency, as it may cause irreversible neurodevelopmental sequelae. Current guidelines, therefore, recommend systematic thyroid function testing (TSH, free T4) in all infants presenting with IHH, regardless of the presence or absence of clinical signs of hypothyroidism [8,9]. Treatment requires levothyroxine at high doses (up to 20-30 µg/kg/day), with close biological monitoring of TSH and free T4, and dose adjustment guided by tumor evolution under propranolol therapy. Normalization of thyroid function constitutes an additional criterion of anti-angiogenic treatment efficacy [8].

Therapeutic management

Propranolol: The Gold-Standard Treatment

The 2008 landmark discovery by Léauté-Labrèze of the efficacy of propranolol in IH [11] constituted a therapeutic revolution. Since then, this non-cardioselective beta-blocker devoid of intrinsic sympathomimetic activity has become the first-line treatment for complicated IH, supplanting systemic corticosteroids previously used despite their numerous adverse effects [6,7].

The mechanism of action of propranolol in IH is multifactorial: immediate vasoconstriction of tumor vasculature (responsible for the rapid color change and softening observed within hours of initiation), inhibition of VEGF synthesis leading to suppression of endothelial and mesenchymal cell proliferation, and induction of endothelial cell apoptosis [6,7,11]. These combined effects account for both the immediate and sustained efficacy of propranolol on IH.

The recommended initiation protocol involves progressive dose escalation over three weeks: 1 mg/kg/day in the first week, 2 mg/kg/day in the second week, and 3 mg/kg/day in the third week, administered in two daily doses at least nine hours apart [6,7]. This gradual escalation aims to minimize the risk of cardiovascular (bradycardia, hypotension), respiratory (bronchospasm during infectious episodes), and metabolic (hypoglycemia, particularly during fasting or gastrointestinal illness) adverse effects. Heart rate and blood pressure monitoring is required at each dose adjustment.

Absolute contraindications to propranolol include sinus bradycardia, second- and third-degree atrioventricular blocks, uncontrolled heart failure, asthma or history of bronchospasm, and arterial hypotension [6,7]. In the setting of PHACES syndrome, the presence of intracranial arterial stenoses constitutes an additional contraindication due to the theoretical risk of cerebral ischemia.

In our case, propranolol was initiated according to this standard protocol, with close monitoring. No notable adverse effects were observed during follow-up. Treatment efficacy was documented at six months by control MRI demonstrating significant regression of hepatic lesions and by parallel normalization of thyroid function.

Alternative Therapeutic Options

Before the propranolol era, high-dose systemic corticosteroids (2-5 mg/kg/day of prednisone for several months) were the reference treatment for complicated IH, with a response rate limited to 30-60% and numerous adverse effects (cushingoid facies, growth retardation, arterial hypertension, hypertrophic obstructive cardiomyopathy, infection susceptibility) [2,4]. In cases of failure, vincristine or interferon alfa could be considered, the latter being associated with a risk of spastic diplegia in infants under one year of age.

In forms of IHH complicated by refractory cardiac failure, hepatic arterial embolization may be proposed to reduce intratumoral vascular flow. Liver transplantation remains an exceptional therapeutic option reserved for diffuse forms resistant to all medical therapies when hepatopathy is severe and irreversible [15]. These radical approaches have become exceedingly rare since the introduction of propranolol.

Follow-Up and Prognosis

Follow-up of IHH under propranolol includes regular clinical monitoring (weight, heart rate, blood pressure, blood glucose), biological surveillance (monthly thyroid function until normalization, then quarterly; liver function tests; full blood count), and morphological monitoring via monthly hepatic ultrasound, complemented by MRI at six months. Treatment is generally continued until 9-12 months of age for medium-sized forms, and beyond for large diffuse forms [6-8].

The overall prognosis of IHH, formerly poor in complicated forms, has been considerably improved by propranolol. Uncomplicated forms follow a course of spontaneous regression, consistent with the natural life cycle of IH, with complete involution in 80% of cases by age seven [1,2]. However, forms complicated by severe cardiac failure or profound hypothyroidism may carry a non-negligible mortality risk in the absence of prompt and appropriate treatment.

## Conclusions

Diffuse IHH is a rare but potentially severe benign vascular tumor of infancy, whose presentation may be misleading in the absence of associated cutaneous hemangiomas. Our observation illustrates several key clinical points worthy of emphasis. Systematic screening for consumptive hypothyroidism by TSH and free T4 assay is mandatory at diagnosis of IHH, regardless of the presence or absence of clinical signs of hypothyroidism. Consumptive hypothyroidism, linked to ID3 overexpression by hemangiomatous tissue, may be profound and requires supra-physiological levothyroxine doses, whose adjustment must be guided by control thyroid function tests and tumor evolution. Multidisciplinary follow-up involving a pediatrician, endocrinologist, cardiologist, and radiologist is essential for optimal management of these infants, enabling early detection and treatment of potential complications and dose adjustment according to morphological and biological evolution.
